# A Case of Primary Uterina Lymphoma Presenting with Bleeding, Pelvic Pain, and Dysmenorrhea

**DOI:** 10.1155/2018/5065738

**Published:** 2018-01-22

**Authors:** Lilian Yukari Miura, Miriam Anyury Daquin Maure, Monica Tessmann Zomer, Reitan Ribeiro, Teresa Cristina Santos Cavalcanti, William Kondo

**Affiliations:** ^1^Vita Batel Hospital, Rua Alferes Angelo Sampaio, 1896 Curitiba, PR, Brazil; ^2^Complejo Hospitalario Metropolitano, Via Simon Bolivar, Panama City, Panama; ^3^Citolab, Rua Vicente Machado 1192 Curitiba, PR, Brazil

## Abstract

Primary non-Hodgkin's lymphoma (NHL) can arise from lymphatic cells located in solid organs (extranodal) and it accounts for 25 to 35% of all NHL. Primary lymphoma on the female genital tract (PLFGT) is a rare disease, comprising 0.2 to 1.1% of all extranodal lymphomas in the female population. In this paper, the authors report an extremely rare case of a 48-year-old woman who exhibited an abnormal uterine bleeding, pelvic pain, and dysmenorrhea history. The transvaginal ultrasound showed an anteverted uterus measuring 153 cm^3^ in volume, with intramural leiomyomas. She underwent a total laparoscopic hysterectomy with bilateral salpingectomy. The histologic evaluation of the specimen showed a follicular lymphoma with diffuse pattern in the endometrium. This report illustrates the difficulty in the diagnosis of primary lymphomas of the female genital tract.

## 1. Introduction

Primary non-Hodgkin's lymphoma (NHL) may arise from lymphatic cells located in solid organs (extranodal) and it accounts for 25 to 35% of all NHL [1–4]. Most of the sites related to this disease are in the gastrointestinal tract and the central nervous system. However, less commonly this pathology may also arise from adrenal glands, thyroid, breasts, bone, prostate, and female genital tract [5–7]. Primary lymphoma on the female genital tract (PLFGT) is a rare disease, which comprises 0.2 to 1.1% of all extranodal lymphomas in the female population [8–10].

The main histological subtypes of non-Hodgkin's lymphoma are the diffuse large B-cell lymphoma, follicular lymphoma, and Burkitt lymphoma [9–11]. Immunohistochemistry studies are important to achieve a correct diagnosis [12–14].

The ovaries are the most common site of PLFGT, followed by the uterine cervix and the uterine body, which is very rare [9, 15]. Usually, in these women the endometrial involvement is secondary to a systemic lymphoma affecting the cervix [15].

## 2. Case Report

A 48-year-old woman, G4 P2 C2, came to our service complaining about abnormal uterine bleeding, pelvic pain, and dysmenorrhea for the last 6 months. She was under clinical treatment for the uterine bleeding with continuous use of hormonal contraceptive (Gestodene 75 mcg + Ethinylestradiol 30 mcg). She had a past surgical history of cholecystectomy, cesarean section, abdominoplasty, and bilateral tubal ligation. The gynecological examination was unremarkable.

On bimanual examination, the uterus was mobile and had a normal size. There was no increase in the volume of the ovaries. Transvaginal ultrasound showed an anteverted uterus, measuring 153 cm^3^, with a heterogeneous pattern, with intramural leiomyomas at the posterior uterine wall. Pap smear was negative for neoplasia.

**Figure 1:**
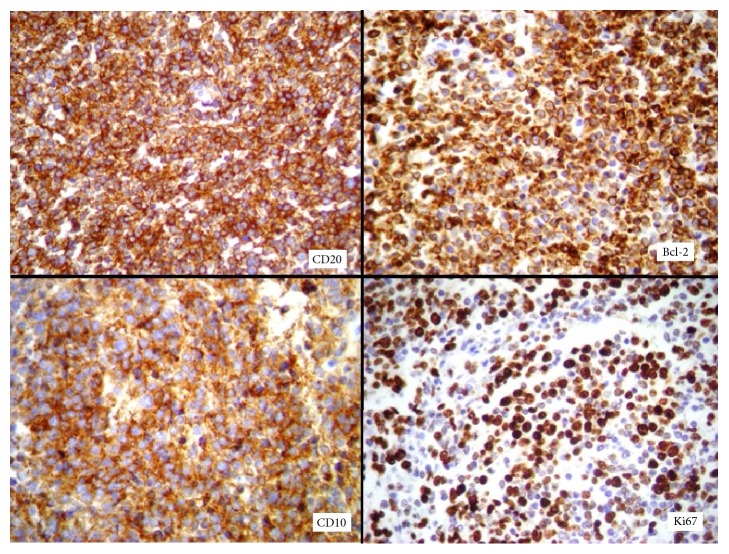
Immunohistochemistry showing positivity of the antibodies CD20, Bcl-2, and CD10 in atypical lymphocytes and Ki67 in 50% of all lymphocytes.

She underwent a laparoscopic hysterectomy with bilateral salpingectomy for a presumed abnormal uterine bleeding and leiomyomas not responding to clinical treatment. The laparoscopic hysterectomy was performed according to the previously reported surgical technique [16]. The surgical procedure was uneventful and lasted 90 minutes. Estimated intraoperative blood loss was 20 cc. The patient was discharged from the hospital, 24 hours after the surgery. The histological evaluation of the specimen identified a dense lymphoid infiltrated in the endometrium, with a diffuse follicular pattern. Immunohistochemical analysis showed CD20, Bcl-2, and CD10 positivity in atypical lymphocytes and Ki67 positivity in 50% of all lymphocytes, leading to a diagnosis of follicular lymphoma with diffuse pattern in the endometrium (Figure 1).

After clinical staging, the disease was diagnosed as follicular lymphoma of the endometrium stage IV, due to the involvement of the bone marrow. The patient was submitted to chemotherapy with six cycles of R-CHOP (rituximab + cyclophosphamide, hydroxydaunorubicin, oncovin, and prednisone), after surgery. Follow-up PET/CT was made sixteen months after surgery and demonstrated increased activity in the retroperitoneum, and it was decided to maintain rituximab for 2 more years.

## 3. Discussion

Lymphomas are malignant tumors that affect the immune system, most commonly, the lymph nodes. However, 40% of the lymphomas are extra nodal [17–20]. When the female genital tract is compromised, which represents 30 to 40% of the cases, it is usually secondary to disseminated disease [21]. Primary lymphoma involving the female genital tract is an uncommon condition, in which the ovaries and cervix are the most frequently affected sites [8–10, 15]. The involvement of extra nodal sites means the worst prognosis [22]. The prognosis usually is analyzed based on the Ann Arbor staging system (Table 1), size and extension of the disease, age, number of nodes affected, LDH level, and lymphoma's grade [20].

It usually occurs in women during the fifth decade of life. Nevertheless, it depends on the histological subtype; as an example, diffuse large B-cell lymphoma is more common between 35 and 45 years old, whereas follicular lymphoma is more frequent in people aged over 50, and Burkitt lymphoma affects children from five to ten years old [23].

The diagnosis is very difficult because of the rarity of this entity. In addition, there is no typical presentation of the symptoms; usually, it depends on the site where the cancer is confined [23]. Some of the symptoms of PLFGT are abnormal uterine bleeding, pelvic pain, abdominal distension, and bloating [17–19]. The “B” symptoms are not frequent in this population, unless the disease has an aggressive behavior along with a large tumor burden [24–26]. In the follicular lymphoma subtype, the clinical presentation usually has an indolent course. Survival rate is very high even without treatment, but it can exhibit a variable clinical presentation, with some patients suffering from aggressive disease [23].

Since clinical evaluation and imaging studies cannot give a definitive diagnosis, most PLFGT is initially treated as being any other common gynecologic malignancy. The definitive diagnosis is obtained after surgery during the pathological examination of the surgical specimen [8]. Immunohistochemistry plays a fundamental role in the characterization of the antibodies and in the classification of the subtypes of lymphomas [27, 28].

The main histological subtype of non-Hodgkin's lymphoma is the diffuse large B-cell lymphoma [9, 11]. The second most common is the follicular lymphoma, followed by Burkitt lymphoma [9]. Immunohistochemical studies are useful to achieve a correct diagnosis, as some low-grade lymphomas (particularly follicular lymphomas and MALT-type lymphomas) are difficult to be distinguished from benign reactive diseases such as severe chronic cervicitis or follicular cervicitis [12–14].

In this case, the patient presented no specific symptoms. She underwent a total laparoscopic hysterectomy for a presumed benign disease (leiomyoma and increased uterine bleeding). The diagnosis of follicular lymphoma with diffuse pattern coming from the endometrium was confirmed after anatomopathological and immunohistochemical evaluations.

The treatment of PLFGT consists of a multimodal approach, including the gynecologist, the clinical oncologist, and the radiation oncologist, trying to individualize each treatment. As this type of lymphoma often compromises premenopausal women, it is very important to consider the patient's fertility. Chemotherapy induces irreversible damage to the ovarian tissue, which leads to premature ovarian failure. This is an important issue that must be discussed with the patient, and the possibility of oocyte or embryo cryopreservation must be remembered in those patients who desire future childbearing [9]. Temporary ovary suppression with GnRH agonists is another alternative, to attempt to reduce the occurrence of premature ovarian failure. However, this procedure is still controversial [29]. Patients who have indication for radiotherapy treatment may be benefited from the ovarian transposition. In this procedure, the ovaries are sutured into the paracolic gutter, in order to attempt to maintain them outside the radiation field, postoperatively [30].

R-CHOP regimen is the standard treatment for follicular lymphoma and includes rituximab plus cyclophosphamide, hydroxydaunorubicin, oncovin (vincristine), and prednisone or prednisolone. The cyclophosphamide is considered a high-risk gonadotoxic drug, oncovin a medium risk [29].

In the posttreatment follow-up, tumor remission is usually evaluated by PET-CT (positron emission tomography-computed tomography) [23].

## 4. Conclusion

PLFGT is a rare condition. Different clinical presentations may lead to a difficult diagnosis, frequently misunderstood during the preoperative setting. Due to the scarce number of cases, there is no formal protocol for the management of PLFGT. Although rare, PLFGT should be considered in the differential diagnosis of organic diseases, leading to increased uterine bleeding and pelvic pain.

## Figures and Tables

**Table 1:** Ann Arbor staging system.

Stage I	Involvement of a single lymph node region (I) or lymphatic structure (Ie)

Stage II	Involvement of 2 or more lymph node regions on same side of diaphragm (II) or with limited, contiguous extralymphatic tissue involvement (IIe)

Stage III	Both sides of the diaphragm are involved and may include spleen (IIIe) or local tissue involvement (IIIe)

Stage IV	Multiple/disseminated involvement of one or more extralymphatic organs, or isolated extralymphatic organ involvement without adjacent regional lymph node involvement, but with disease in distant site (s), or any involvement of the liver, bone marrow, pleura, or cerebrospinal fluid (i.e., bone marrow)

(A) or (B)	Designates absence/presence of “B” symptoms

(E)	Localized, solitary involvement of extra lymphatic tissue, excluding liver and bone marrow
